# Detection and Quantification of CWD Prions in Fixed Paraffin Embedded Tissues by Real-Time Quaking-Induced Conversion

**DOI:** 10.1038/srep25098

**Published:** 2016-05-09

**Authors:** Clare E. Hoover, Kristen A. Davenport, Davin M. Henderson, Laura A. Pulscher, Candace K. Mathiason, Mark D. Zabel, Edward A. Hoover

**Affiliations:** 1 Prion Research Center, Department of Microbiology, Immunology, and Pathology, College of Veterinary Medicine and Biomedical Sciences, Colorado State University, Fort Collins CO 80523, USA

## Abstract

Traditional diagnostic detection of chronic wasting disease (CWD) relies on immunodetection of misfolded CWD prion protein (PrP^CWD^) by western blotting, ELISA, or immunohistochemistry (IHC). These techniques require separate sample collections (frozen and fixed) which may result in discrepancies due to variation in prion tissue distribution and assay sensitivities that limit detection especially in early and subclinical infections. Here, we harness the power of real-time quaking induced conversion (RT-QuIC) to amplify, detect, and quantify prion amyloid seeding activity in fixed paraffin-embedded (FPE) tissue sections. We show that FPE RT-QuIC has greater detection sensitivity than IHC in tissues with low PrP^CWD^ burdens, including those that are IHC-negative. We also employ amyloid formation kinetics to yield a semi-quantitative estimate of prion concentration in a given FPE tissue. We report that FPE RT-QuIC has the ability to enhance diagnostic and investigative detection of disease-associated PrP^RES^ in prion, and potentially other, protein misfolding disease states.

Prion diseases, or transmissible spongiform encephalopathies, are fatal neurodegenerative diseases that include Creutzfeldt-Jakob disease (CJD) in humans, bovine spongiform encephalopathy (BSE), sheep scrapie, and cervid chronic wasting disease (CWD). Disease is initiated when a misfolded isoform (designated PrP^Sc^, PrP^RES^, PrP^CWD^, or PrP^D^) induces templated misfolding of the normal cellular prion protein (PrP^C^) to propagate via a process of seeded polymerization^1^,^2^. Prions are characterized by increased formation of β sheets, propensity to aggregate into amyloid fibrils, and resistance to protease and acid digestion^3^,^4^,^5^. Typical histologic lesions include spongiform neuropathology and neurodegeneration associated with deposition of PrP^Sc^, and reactive astrogliosis^4^.

Definitive diagnosis of CWD traditionally relies on detection of PrP^CWD^ by immunohistochemistry (IHC), western blot (WB), enzyme-linked immunosorbent assay (ELISA), or animal bioassay. The tissues tested typically include antemortem biopsies of tonsil or rectal associated mucosal lymphoid tissue (RAMALT) or postmortem sampling of the obex region of the brainstem. Analysis by these techniques requires a minimum of two separate tissue samples, formalin fixed tissues for IHC and fresh or frozen tissues for WB and ELISA that can create result disparity in results if PrP^CWD^ is not homogenously distributed or at low levels. While immunodetection methods can provide qualitative estimation of prion concentration, calculating the infectious titer of a prion tissue sample requires an end-point dilution bioassay, which relies on disease incubation time to determine infectivity levels. Bioassay limitations include animal availability, cost, and time required (often greater than one year to complete). Cell culture based assays have been developed to supplant animal bioassays^6^,^7^, but remain relatively time consuming, may be limited to specific prion strains, and are not suitable for fixed tissue samples.

Previous studies have established protocols for the isolation of disease associated prion proteins from formalin-fixed paraffin-embedded tissue sections^8^,^9^,^10^. Transgenic mice expressing hamster PrP^C^ intracerebrally inoculated with prions isolated from formaldehyde fixed paraffin-embedded 263K scrapie strain terminal brains developed clinical disease^8^. Prion protein can be demonstrated in some archived formalin-fixed paraffin-embedded tissues and detected by western blotting, albeit with lower levels of sensitivity, typically limiting this method to terminal samples^9^,^10^. A separate protein blotting technique, the paraffin embedded tissue blot, can detect PrP^D^ deposition in the brain of pre-clinical animals by using harsh epitope retrieval and immunostaining; however, the multi-step process is labor intensive and cannot provide quantitative data^11^.

In most samples collected prior to terminal disease, PrP^CWD^ levels are often below the threshold of detection by IHC or WB; thus detection at these time points requires *in vitro* amplification methods for detection^12^,^13^,^14^,^15^. One amyloid amplification method is real time quaking induced conversion (RT-QuIC), which utilizes templated prion seeded conversion of bacterially expressed recombinant PrP^C^ into amyloid structures that are measured by thioflavin T (ThT) fluorescence^15^. Repeated cycles of shaking are used to exponentially increase initial low levels of PrP^CWD^, potentially facilitating a greater sensitivity of detection. In addition, RT-QuIC amyloid formation kinetics have been adapted to estimate the low quantity of prions in samples of body fluids and excreta^16^,^17^ with a sensitivity approaching bioassay^14^.

Here, we describe a simple and rapid technique to detect and quantify PrP^CWD^ prion seeding activity from fixed paraffin-embedded (FPE) tissues by RT-QuIC. Assessment of RT-QuIC amyloid formation rate kinetics enabled quantification of PrP^CWD^, providing a useful estimation of prion infectivity in IHC positive tissues. The combination of IHC and FPE RT-QuIC makes possible the description of PrP^CWD^ distribution and estimation of the infectious titer in tissues, including those with minimal or absent PrP^CWD^ detection by IHC.

## Results

### Detection of PrP^CWD^ in FPE brain samples

**Figure 1:**
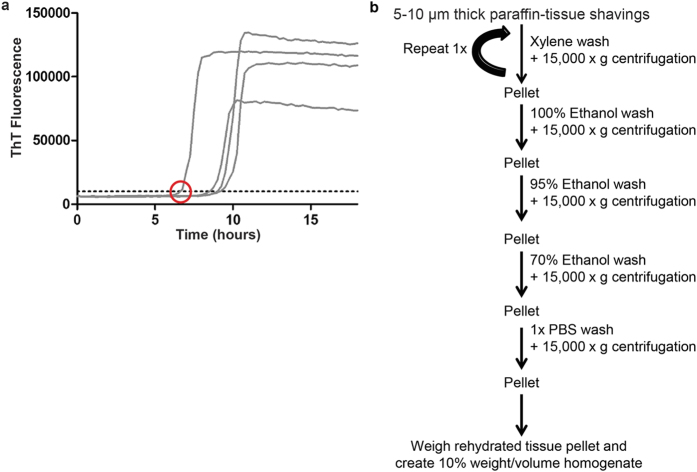
Overview of methods. (**a**) Representative RT-QuIC fluorescence curve. As prion-enciphered amyloid is formed out of rPrP^C^ substrate the RT-QuIC reaction transitions from lag phase to exponential phase. Amyloid formation is measured through thioflavin T (ThT) fluorescence emission as it binds amyloid. The Ct value is defined as the time (hours) for a positive RT-QuIC reaction to reach a predefined threshold (horizontal line), indicated by the red circle. The rate of amyloid formation is calculated as the inverse of Ct (1/h). (**b**) Schematic of FPE protein extraction method.

RT-QuIC is a prion-seeded amyloid amplification assay that uses recombinant PrP^C^ as the substrate and thioflavin T (ThT) amyloid-binding fluorescence emission as the indicator of prion enciphered amyloid fibril formation^18^. RT-QuIC generation of prion seeded amyloid fibril formation follows a logarithmic pattern, with an early lag phase below the level of ThT detection and a rapid exponential phase as amyloid fibrils are formed, analogous to DNA replication in PCR reactions (Fig. 1a). A positive RT-QuIC reaction is defined as when a ThT fluorescence curve crosses an established baseline threshold, calculated to be five standard deviations above the average initial fluorescence. The time for a reaction to reach the threshold is defined as the C_t_ value (hours). Since ThT fluorescence is recorded in real time, the rate of amyloid formation can be calculated as the inverse of the C_t_ value (1/h). Previous studies have demonstrated a direct, linear relationship between the C_t_ value and concentration of prions in the sample used to seed a reaction. Thus, samples with larger prion burdens have greater amyloid formation rates^14^,^16^.

**Figure 2:**
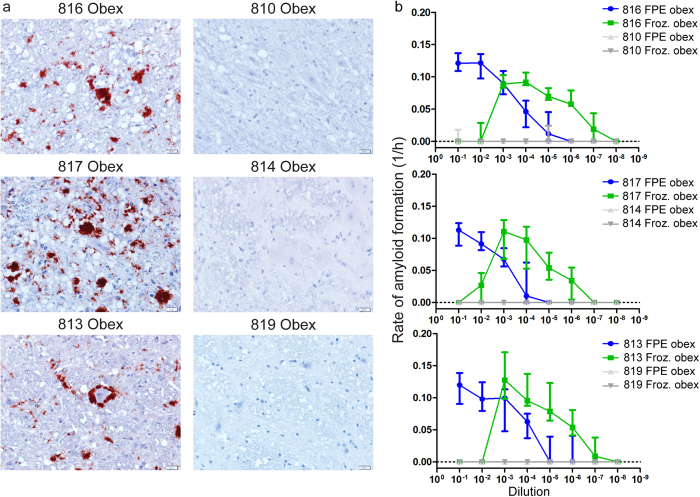
Detection of amyloid seeding activity in FPE obex tissue. (**a**) Immunohistochemistry of obex from three terminal white-tailed deer. All display variably sized PrP^CWD^ plaque accumulations in the neuropil. The corresponding negative control animals do not display any PrP^CWD^ immunoreactivity. Images are 400× magnification, measurement bars represent 20 μ m. (**b**) RT-QuIC rates of amyloid formation of serial dilutions of FPE tissues from IHC positive obex and frozen 10% obex tissue homogenate. FPE samples from IHC positive obex are detectable in RT-QuIC from all three white-tailed deer examined. The RT-QuIC linear range in FPE obex samples extends from 10^−1^ to 10^−6^ whereas the linear range in frozen 10% homogenates is from 10^−4^ to 10^−7^. Spontaneous amyloid formation from rPrP^C^ negative control samples occurred equally rarely in both FPE and frozen homogenates. Each point represents median and interquartile range (IQR) and is derived of 8 replicates from a minimum of two separate experiments.

To determine if our FPE protein extraction method preserved PrP^CWD^ amyloid seeding activity for RT-QuIC detection, we selected the obex portion of the medulla oblongata region of the brainstem from white-tailed deer (WTD) infected with CWD by an aerosol route. This region had marked PrP^CWD^ deposition demonstrated by IHC (Fig. 2a)^19^. Obex samples from sham-inoculated WTD were processed in parallel as negative controls. Microtome paraffin shavings of 8 to 10 μ m thickness from FPE tissue blocks were treated with a series of xylene and alcohol washes to remove paraffin and rehydrate the tissue sections consistent with conventional histologic section processing. Following tissue rehydration, extracted samples were weighed and converted into a 10% weight per volume homogenate to standardize the amount of sample analyzed by RT-QuIC (Fig. 1b).

Serial dilutions from 10^−1^ to 10^−8^ were analyzed comparing the FPE-extracted obex sample homogenates to the corresponding unfixed, frozen obex sample homogenates collected at necropsy from the same animal. All WTD inoculated with CWD had marked, variably sized PrP^CWD^ plaque accumulation and neuropil vacuolation in the obex by IHC consistent with terminal disease pathology (Fig. 2a). No PrP^CWD^ immunoreactivity was observed in the corresponding negative control obex samples. RT-QuIC ThT fluorescence readings were converted to rates of amyloid formation by calculating the C_t_ value. The dilutional series data demonstrated that the FPE obex homogenates maintained prion amyloid seeding activity over a 10^−1^ to 10^−7^ dilution range (Fig. 2b). By comparison, dilution of parallel frozen unfixed obex homogenates from the same animal contained prion seeding activity from dilutions 10^−3^ to 10^−8^. The absence of RT-QuIC amyloid seeding activity in 10^−1^ and 10^−2^ dilutions of unfixed frozen samples is well documented and believed to be the result of inhibitors present in high concentrations in nervous tissue homogenates, which must be diluted before amyloid formation can occur^20^. Background, spontaneous unseeded amyloid formation from rPrP^C^ substrate was rare in negative control obex homogenates derived from either FPE or unfixed frozen obex samples and comparable between the two sample preparation techniques. These results demonstrated that the FPE RT-QuIC protocol can consistently and specifically detect PrP^CWD^ amyloid seeding activity in FPE obex homogenates from CWD-infected, but not control, WTD.

### Detection of PrP^CWD^ seeding activity in FPE retropharyngeal lymph node

**Figure 3:**
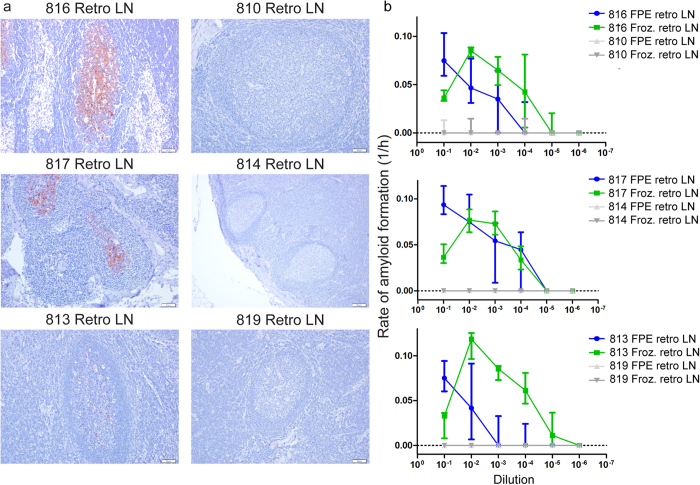
Detection of amyloid seeding activity in FPE retropharyngeal lymph node. (**a**) Immunohistochemistry of retropharyngeal lymph nodes from three terminal white-tailed deer. Mild to marked PrP^CWD^ immunoreactivity is present in germinal centers of lymphoid follicles. No PrP^CWD^ immunoreactivity is detected in negative control animals. All IHC images are 200× magnification, measurement bars represent 20 μ m. (**b**) RT-QuIC of serial dilutions of terminal-disease, IHC positive FPE retropharyngeal lymph node and 10% frozen homogenate. Similar to obex samples (see Fig. 2), RT-QuIC successfully detected amyloid seeding activity in FPE retropharyngeal lymph node samples. The range of detection was similar between FPE samples and frozen samples, generally from 10^−1^ to 10^−5^. Spontaneous amyloid formation was rare in negative controls and did not differ between FPE and frozen samples. Each point represents median and IQR and is derived of 8 replicates from a minimum of two separate experiments.

Next, we examined retropharyngeal lymph nodes from the same WTD analyzed in Fig. 2 using identical FPE protein extraction methodology. Retropharyngeal lymph nodes from WTD inoculated with CWD displayed moderate to marked PrP^CWD^ deposition in germinal centers of lymphoid follicles by IHC consistent with a CWD disease state (Fig. 3a). By comparison, retropharyngeal lymph nodes from corresponding negative control WTD did not display any PrP^CWD^ immunoreactivity. Both FPE and frozen lymph node homogenates displayed amyloid seeding activity over dilutions 10^−1^ to 10^−5^ revealing lower endpoints and less inhibitor activity when compared to nervous tissue (Fig. 3b). These results confirmed that the FPE protein extraction protocol is applicable to demonstrating amyloid seeding activity in FPE lymphoid tissues of WTD.

### Sensitivity of RT-QuIC prion seeding activity detection in FPE tissues

**Figure 4:**
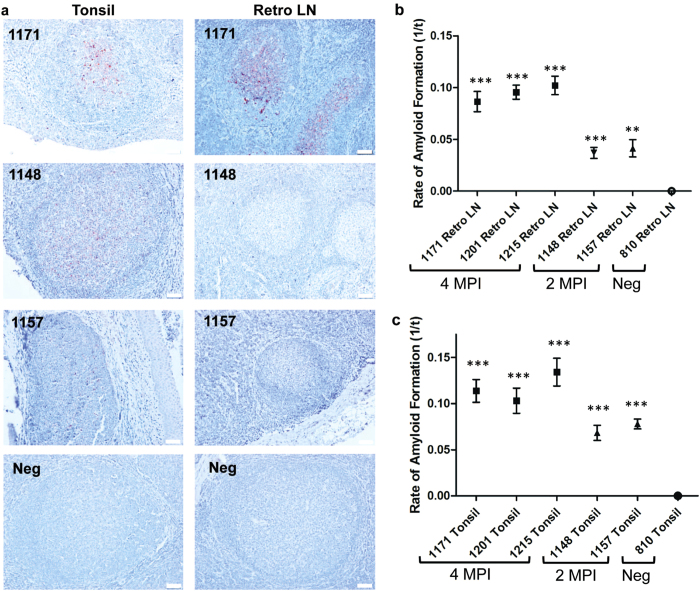
Analysis of FPE lymphoid tissues from subclinical CWD-infected WTD by RT-QuIC. (**a**) Representative IHC of FPE lymphoid tissues from subclinical, CWD-infected WTD. Tissues collected at 4 MPI have increased density of PrP^CWD^ staining when compared to 2 MPI tissues reflecting a greater prion accumulation over the disease course. The low-level of PrP^CWD^ staining at 2 MPI reflects the early course of disease. RT-QuIC detected amyloid seeding activity in FPE samples that are IHC negative (1148 Retro LN). All IHC images are 200× magnification, measurement bars represent 50 μ m. (**b**) RT-QuIC of subclinical WTD retropharyngeal lymph nodes. All retropharyngeal lymph nodes from subclinical animals displayed significant amyloid seeding activity (means analyzed by one sample t-test; **p <  0.01, ***p <  0.001) when compared with respective negative control tissues. Lymph nodes collected at 4 MPI had higher RT-QuIC amyloid formation rates than tissues collected at 2 MPI which is consistent with greater PrP^CWD^ amyloid seeding activity. Each point represents the mean and SEM derived from eight replicates and in two separate experiments. All FPE samples are assayed at a 10^−1^ dilution. (**c**) RT-QuIC of subclinical WTD tonsils. All tonsils from subclinical animals displayed significant amyloid seeding activity (means analyzed by one sample t-test; ***p <  0.001) when compared with respective negative control tissues. Similar to the retropharyngeal lymph nodes in (**b**), tonsils collected at 4 MPI had higher RT-QuIC amyloid formation rates than tissues collected at 2 MPI which is consistent with greater PrP^CWD^ amyloid seeding activity. Each point represents the mean and SEM derived from eight replicates and in two separate experiments. All FPE samples are assayed at a 10^−1^ dilution.

We sought to estimate the sensitivity of FPE RT-QuIC seeding and concurrently determine whether prion-related amyloid seeding could be detected in IHC-negative tissues from CWD inoculated WTD due to RT-QuIC amplification of initial prion seeds. We selected tonsil and retropharyngeal lymph node samples from subclinical WTD that were orally inoculated with CWD and sacrificed at either two or four months post-inoculation (MPI). At these collection times, PrP^CWD^ was variably detectable by IHC in retropharyngeal lymph nodes and tonsils (summarized in Table 1). At 2 MPI, minimal or no PrP^CWD^ immunoreactivity was observed in tissues and was characterized by faint granular staining in germinal centers and limited lymphoid follicle distribution (Fig. 4a). By contrast, at 4 MPI both lymphoid tissues displayed strong PrP^CWD^ IHC immunoreactivity with dense granular staining affecting the majority of the follicles. FPE RT-QuIC of tonsil and retropharyngeal lymph nodes from both subclinical time points (2 and 4 MPI) demonstrated amyloid seeding activities that were significantly different than corresponding FPE negative control tissues (One sample t-test, p >  0.01) (Fig. 4b). Tissues from 2 MPI had lower rates of amyloid formation, correlating with the minimal or absent PrP^CWD^ deposition observed by IHC. Likewise, higher amyloid formation rates were present in 4 MPI tissues, correlating with the marked PrP^CWD^ distribution noted by IHC. There were no significant differences between the amyloid formation rates of tonsil or retropharyngeal lymph nodes collected at the same MPI from WTD (p >  0.05, 2 MPI one sample t-test, 4 MPI one-way ANOVA). These results demonstrated correlation of FPE RT-QuIC amyloid formation rates with PrP^CWD^ detection by IHC. Additionally, we detected prion-related amyloid seeding activity in FPE samples at or below the threshold of IHC detection at 2 MPI.

### Correlation of RT-QuIC reaction rate with bioassay

**Figure 5:**
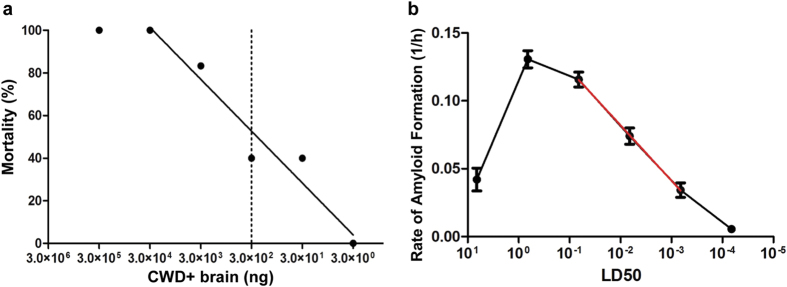
Endpoint dilution bioassay and RT-QuIC quantification of a CWD + brain sample. (**a**) Percent mortality of cervidized transgenic mice that developed terminal CWD. The dashed vertical line indicates the LD_50_ which is calculated to be a 10^−5^ dilution or 3.33 ×  10^6^ LD_50_/g of brain tissue. (**b**) RT-QuIC rates of bioassay inoculum dilutional series. The green solid curve represents the RT-QuIC amyloid formation rates (in LD_50_). The linear range extended from 10^−4^ to 10^−6^. A semi-fit log line was extrapolated from the linear range and used to relate y (rate of amyloid formation) to x (LD_50_). The equation of this line, in the form y =  m log (x) +  b, was used for FPE quantification displayed in Table 2. Each point represents the mean and SEM of 48 replicates derived from eight separate experiments.

To establish a biological correlation between the amyloid formation rate and an estimated tissue prion burden, we analyzed the RT-QuIC rate from a dilutional series of a deer brain homogenate that was assayed by cervid PrP^C^ transgenic mouse end-point dilutional bioassay to yield a calculated prion titer. The bioassay inoculum was a brain homogenate pooled from six terminal experimentally inoculated WTD (cervid brain pool 6; CBP6) that were confirmed PrP^CWD^ positive by western blot prior to pooling. Transgenic mice engineered to express the cellular cervid prion protein were inoculated intracerebrally with a dilution of the CBP6 extending from 1.0% to 0.00001% weight per volume (Fig. 5a). Using Reed-Muench calculations, the mouse bioassay LD_50_ was calculated to be a 10^−5^ dilution, the equivalent of 3.33 ×  10^6^ LD_50_/gram of brain tissue^21^. The CBP6 dilutional series linear range of RT-QuIC amyloid formation rates extended from 10^−4^ to 10^−6^ and was fit with a semi-log linear regression line (Fig. 5b). The equation of the semi-log fit line relates RT-QuIC amyloid formation rates on the y-axis to LD_50_ on the x-axis, enabling semi-quantitative estimation of the prion titer for a sample.

### Quantification of prion-related seeding activity in FPE tissues

The correlation of FPE- RT-QuIC rates with PrP^CWD^ immunoreactivity in pre-clinical WTD (Fig. 4) led us to estimate prion seeding activity in FPE samples by extrapolation to the RT-QuIC reaction rate standard curve derived from a bioassayed CWD brain pool as previously described^17^. To establish the standard curve, we used a semi-log linear regression equation defined as: amyloid formation rate =  0.04073 log (sample LD_50_) +  0.1633. Based on the RT-QuIC amyloid formation rate we were able to calculate the LD_50_ equivalents in each gram of FPE tissue using the defined equation^16^. For example, the estimated PrP^CWD^ prion seeding activity in 1 gram of FPE tonsil from a 4 MPI WTD is calculated to be 3039.6 LD_50_. The estimated LD_50_ per gram of FPE tissue in the subclinical WTD samples analyzed in Fig. 4 are listed in Table 2. By correlating IHC detection and the RT-QuIC seeding rates in the FPE samples tested, we were able to consistently detect moderate IHC staining in samples with greater than 200 LD_50_/g of FPE tissue. When comparing PrP^CWD^ detection by IHC and RT-QuIC, we determined that RT-QuIC could detect as little as 39 LD_50_/g of tissue whereas the apparent threshold for detection by IHC examination was 50 LD_50_/gram. This indicated that FPE RT-QuIC had a greater sensitivity than IHC alone for detecting CWD prion infection; however, a larger data set is needed to make a more definitive comparison and quantify the differences in sensitivity. Estimates of prion burdens in FPE samples also enable biological characterization of early CWD prion infection previously unavailable using IHC analysis alone. For example, FPE tissues collected during subclinical CWD infection (2 MPI to 4 MPI) demonstrated that tonsils consistently accumulated a higher CWD prion burden when compared to retropharyngeal lymph nodes. Likewise, the lymphoid tissues examined at 4 MPI contained at least a 10-fold greater LD_50_/gram PrP^CWD^ deposition in the same tissues on average when compared to 2 MPI. These observations suggest interesting insights into the early pathogenesis of CWD; however, a larger sample size is necessary to draw definitive conclusions.

## Discussion

We describe a method for detection and semi-quantification of prion amyloid seeding activity in microtome sections of fixed paraffin-embedded tissues. Consistent with previous formalin fixed paraffin-embedded tissue studies analyzing prions by western blot or paraffin-embedded tissue blot we demonstrate that prion amyloid seeding is maintained throughout the tissue aldehyde cross-linking, dehydrating, lipid extracting, and paraffin embedding histological processes^9^,^11^. The FPE RT-QuIC method makes possible the analysis of archived samples when only fixed paraffin-embedded tissues are available and minimizes the sampling differences encountered when separate fixed and frozen tissue samples are collected and examined. The additional semi-quantitative information provided by FPE RT-QuIC allows more precise investigations into PrP^CWD^ accumulation by tissues compared with subjective IHC scoring of mild/moderate/marked or lymphoid follicle counts. We have demonstrated the application of this method with a small observational study of deer early in their CWD disease progression.

An interesting observation of FPE RT-QuIC analysis of obex samples in Fig. 2 was a shift in the range of detection. Previous RT-QuIC studies of frozen brain samples have consistently demonstrated an inability to detect seeding activity in 10^−1^ to 10^−3^ dilutions^16^,^20^. This is presumed to be due to inhibitors present in brain homogenates that are diluted out or minimized in smaller dilutions, permitting the amyloidogenesis from rPrP^C^ reaction to occur. Investigations identifying the biochemical nature of these amyloid seeding inhibitors in brain are ongoing.

The correlation between IHC deposition, RT-QuIC amyloid seeding rate, and the disease course expressed as MPI is clear. Small differences in tissue amyloid seeding rates between animals collected at the same time point are expected due to biological variability. Importantly, detection of PrP^CWD^ seeding activity in lymphoid tissues of preclinical WTD with no or minimal detectable PrP^CWD^ by IHC suggests either greater sensitivity provided by RT-QuIC amplification or the presence of prion conformers that are sensitive to protease treatments used during IHC to abolish PrP^C^ background^22^. Likewise, protein misfolding cyclic amplification analyses of some tissues in CWD-infected WTD have shown a contrast between amplifiable prion enciphering activity and detection of PrP^CWD^ in tissues by IHC^12^. Further analysis with a larger data set is needed to accurately compare the diagnostic detection sensitivities and specificities of RT-QuIC and IHC. As with IHC, the degree to which the amyloid seeding activity measured by RT-QuIC is infectious is unknown. Here, we report on FPE RT-QuIC as a means to identify prion-derived amyloid seeding activity in fixed tissue and use this information to detect prion infection. This information can then be used, if desired, to estimate tissue prion burden by extrapolation to a bioassayed reference sample.

Building on previous work, we used the mathematical relationship between PrP^CWD^ seed concentration and the amyloid formation rate to estimate PrP^CWD^ concentration in tissues^16^,^17^. The RT-QuIC quantification method we applied is consistent with previous literature that based quantification on endpoint dilution^14^ and quantitative PMCA that demonstrates initial PrP^CWD^ seed determines the amplification round of detection^23^. In addition, the IHC staining results correlated well with RT-QuIC rates, further validating the relationship between amyloid seeding rates and concentration of prions in the sample. The RT-QuIC amyloid seeding reaction, which is consistent across various sample types, requires the presence of a conversion-competent prion seed to initiate amyloid formation. Bioassay data provides information on the lethality of the tested sample that is presumed to correspond to the prion burden of the inoculum. Thus, we can estimate the prion burden and lethality of a sample, such as FPE tissues, by extrapolating to the bioassay data. Of course this remains an approximation since the relationship of seeding activity and infectivity would be expected to vary with tissue, prion strain, RT-QuIC substrate sensitivity, and inhibitors that may be present, especially at low dilutions. Additional studies are needed to more closely quantify the infectivity and lethality of FPE RT-QuIC seeds and amyloid products. Frozen lymphoid tissue homogenates likely contain a variety of prion aggregation states as has previously been demonstrated in brain homogenates^24^,^25^. It is not known whether this aggregation diversity is maintained throughout the FPE extraction process or if a single aggregate type or conformation is preferentially being selected. We included multiple experiments and replicates in our RT-QuIC analysis to mitigate uneven distribution of prion seeds in our samples and assay variability. It is also plausible that a small fraction of prion content is lost through the FPE extraction process. The one log lower upper limit of detection in FPE brain and retropharyngeal lymph nodes (Figs 2 and 3) could reflect these factors. If this is the case, our mathematical calculations may be underestimating the true prion seeding concentration. Despite these limitations, the FPE RT-QuIC quantification method still provides a useful means of estimating prion burden in diverse tissues when animal bioassays are precluded by large sample numbers or financial feasibility.

There is little to no evidence at present that RT-QuIC generates infectious prions. However, Sano *et al.* have reported bioassay of amyloid fibrils generated by the first round of RT-QuIC reactions seeded with infectious brain homogenate induced prion-specific disease when inoculated into wild-type mice^26^. However, amyloid products from additional rounds of RT-QuIC did not demonstrate any infectivity. Lack of infectivity in amyloid fibrils generated from recombinant PrP^C^ is not unique to RT-QuIC. Amyloid fibrils produced by protein misfolding cyclic amplification using recombinant PrP^C^ substrate require polyanionic cofactors, such as RNA or specific lipids, to demonstrate infectivity in mouse bioassay^27^,^28^. Possible reasons for lack of infectivity of RT-QuIC products include the use of truncated rPrP^C^ as the substrate and absence of post-translational modifications, including glycosylation^27^.

In summary, we present a rapid and simple method of PrP^CWD^ extraction from fixed paraffin embedded tissues and analysis for prion amyloid seeding activity using RT-QuIC. We believe this method can facilitate quantitative and correlative investigations of kinetics and pathogenesis during early and carrier states of prion diseases and potentially other protein misfolding disorders.

## Materials and Methods

### Animals

Hand-raised, indoor-adapted white-tailed deer (WTD) (*Odocoileus virginianus*) were provided through long-standing collaboration of David Osborn, Sallie Dahmes, Karl Olsen, and Robert Warren, at the Warnell School of Forestry and Natural Resources, University of Georgia. Deer were housed in an indoor research facility at Colorado State University. Institutional Animal Care and Use Committee (IACUC) protocols for animal treatment and handling were followed. All animal studies were approved by Colorado State University IACUC. Archived tissues from CWD-infected WTD were used to optimize these techniques. All WTD with 800-series ID numbers were intranasally inoculated with two 1 mL doses of a 5% CWD positive brain homogenate^19^. Deer were allowed to develop clinical disease and tissues were collected at terminal necropsy. All deer with 11-series and 12-series ID numbers were inoculated *per os* with 0.5 grams of a CWD positive pool of cervid brain. Deer were sacrificed, necropsied, and tissues collected at scheduled monthly intervals post-inoculation prior to development of clinical signs associated with CWD infection.

### Tissue processing

At necropsy, all tissues were individually collected with a prion-free instruments to prevent cross contamination and were divided in half with one half stored at −80 °C until use and the other half fixed for a minimum of 48 hours in periodate-lysine-paraformaldehyde (PLP) fixative. After 48 hours tissues were transferred to sterile 1 ×  PBS until trimming and then stored in 70% ethanol. Sections of the obex region of the medulla oblongata, retropharyngeal lymph node, and tonsil were trimmed and subjected to routine paraffin embedding. Frozen tissues were homogenized in 1 × PBS at 10% weight per volume with a Blue Bullet^TM^ bead-beater homogenizer (Next Advance) with care to avoid any cross contamination among tissue homogenates.

### Protein extraction from FPE blocks

Eight to 10 μ m thick paraffin-embedded tissue wax curls were cut from paraffin blocks using a microtome (Leica) with a prion-free microtome blade and prion-free forceps for every FPE sample to avoid cross contamination. Paraffin-tissue curls were subjected to a series of xylene and graded alcohol washes (100%, 95%, and 70%) to remove paraffin and rehydrate tissues similar to standard histological methods. Following alcohol treatments rehydrated FPE tissues were washed with 1 × PBS. Rehydrated FPE tissue was homogenized in at 10% weight per volume by manual disruption or a bead homogenizer as described above.

### Immunohistochemistry for PrP^CWD^

Following routine paraffin embedding, 5 μ m microtome tissue sections were placed on positively charged glass slides. Tissues were deparaffinized, rehydrated with graded alcohols, and treated with 88% formic acid digestion prior to heat-induced epitope antigen retrieval in the 2100-Retriever^TM^ (Prestige Medical). Following retrieval, endogenous peroxidase activity was quenched prior to prion epitope detection with anti-prion antibody BAR224 (Caymen chemical) at 2 μ g/mL concentration and Envision+^TM^ anti-mouse HRP-labeled polymer (Dako). Following antibody treatment, antigen was visualized with 3-amino-9-ethylcarbazole (AEC) substrate chromogen (Dako). Tissue sections were counterstained with Meyer’s hematoxylin (Dako) and 0.1% bicarbonate bluing reagent prior to coverslipping with aqueous mounting media (Dako).

### Recombinant Syrian hamster PrP^C^ protein purification

Purification was performed as previously described^14^,^16^,^29^. Briefly, recombinant truncated Syrian hamster PrP^C^ (90–231) (SHrPrP) was expressed and purified from BL21 Rosetta (Novagen) *Escherichia coli*. Cells from a glycerol stock were cultured at 37 °C in lysogeny broth (LB) media with selection antibiotics kanamycin and chloramphenicol until the final OD_600_ was at least 2.5. Cell lysis was performed using Bugbuster^TM^ reagent, supplemented with Lysonase^TM^ (EMD Biosciences) and inclusion bodies harvested by centrifugation at 15,000 ×  g. Inclusion bodies were dissolved in solubilization buffer (8 M guanidine hydrochloride, 100 mM Na_2_HPO_4_) and applied to NiNTA superflow resin (Qiagen) that had been equilibrated with denaturation buffer (6 M guanidine hydrochloride, 100 mM Na2HPO_4_, 10 mM Tris). The resin-SHrPrP was loaded on to a XK16–60 column (GE Healthcare) and purified using a Bio-Rad Duoflow^TM^ FPLC. A gradient from denaturation buffer to refolding buffer (100 mM Na_2_HPO_4_, 10 mM Tris) was applied to induce protein refolding prior to a wash with refolding buffer. Refolding was followed by a gradient from refolding to elution buffer (100 mM NaH_2_PO_4_, 10 mM Tris, 0.5 M imidazole) and all fractions were collected. The fractions from the elution peak were pooled and dialyzed (20 mM NaH_2_PO_4_) overnight. Protein concentration was calculated by measuring the A_280_ and using a coefficient of extinction of 25,900 in Beer’s Law.

### RT-QuIC assay

Assay conditions were as previously described^16^,^17^. FPE tissue homogenates or 10% frozen tissue homogenates were diluted in 0.1% sodium dodecyl sulfate (SDS)/1 ×  PBS buffer as described. For subclinical WTD samples, a 10^−1^ dilution of FPE homogenates was used to seed the RT-QuIC reaction. Two μ L of diluted sample was added to the RT-QuIC reaction buffer consisting of 20 mM NaH_2_PO4, 320 mM NaCl, 1.0 mM EDTA, 1 mM Thioflavin T (ThT) and 0.1 mg/mL SHrPrP. RT-QuIC reactions were carried out in black, optical bottom 96-well plates (Nunc) in a BMG Labtech Polarstar^TM^ fluorometer. RT-QuIC cycles consisted of 1 minute of shaking at 700 rpm followed by 1 minute of rest, repeated for 15 minutes. Fluorescence was read at the conclusion of each 15 minute shake/rest cycle with an excitation of 450 nm and emission of 480 nm, gain of 1700, and each well was measured with 20 flashes per well with an orbital averaging of 4. Each RT-QuIC assay was performed for 250 cycles, the equivalent of 62.5 hours.

### CWD bioassay in transgenic cervidized mice

All mice were bred and maintained at Colorado State University Lab Animal Resources, accredited by the Association for Assessment and Accreditation of Lab Animal Care International in accordance with protocols approved by the Institutional Animal Care and Use Committee at Colorado State University. All animal studies were approved by Colorado State University IACUC. Transgenic mice overexpressing the cervid cellular prion protein were inoculated with 30 μ L of a homogenate pooled from six terminal experimentally inoculated WTD brain samples (cervid brain pool 6; CBP6) diluted from 1.0% to 0.00001% concentration. Mice were observed for clinical signs and sacrificed when they reached terminal disease. Mice that did not develop clinical signs were sacrificed at 500 days post inoculation when the study was terminated. Brains were harvested from all animals at time of sacrifice and analyzed for PrP^CWD^ by immunohistochemistry following the above protocol or western blot^30^.

### RT-QuIC rate analysis and quantification

The time to threshold, or C_t_ values, were calculated by determining the time (hours) for a positive amyloid seeding reaction to reach a predetermined threshold (established as 5 standard deviations above the average baseline fluorescence of all samples). The rate of amyloid formation was calculated as the inverse of the Ct value (1/h). FPE RT-QuIC amyloid formation rates for each sample were confirmed to be normally distributed using the D’Agostino-Pearson normality test. RT-QuIC amyloid formation rates were statistically compared with corresponding negative controls using a one-sample t test. An equation relating the amyloid formation rate (1/h) to the LD_50_ for mouse bioassayed material (CBP6) was created by applying a best-fit line to the linear range, 10^−4^ to 10^−6^, of the RT-QuIC CBP6 dilution. This equation, in the format of y =  m log (x) +  b, was used to correlate LD_50_ data (x) to RT-QuIC rates (y).

## Additional Information

**How to cite this article**: Hoover, C. E. *et al.* Detection and Quantification of CWD Prions in Fixed Paraffin Embedded Tissues by Real-Time Quaking-Induced Conversion. *Sci. Rep.* **6**, 25098; doi: 10.1038/srep25098 (2016).

## Figures and Tables

**Table 1:** Summary of PrP^CWD^ IHC immunoreactivity in lymphoid tissues of subclinical WTD.

WTD	Tonsil	Retropharyngeal LN
1171 (4 MPI)	104/129	70/112
1201 (4 MPI)	146/183	177/232
1215 (4 MPI)	151/191	183/216
1148 (2 MPI)	8/134	0/95
1157 (2 MPI)	5/125	1/99

The number of lymphoid follicle germinal centers with PrP^CWD^ immunoreactivity out of the total number of follicles in the sections examined. This gives a general comparison of how much PrP^CWD^ is present in the tissue by IHC analysis.

**Table 2:** The estimated LD_50_/gram of FPE samples.

WTD	Tonsil	Retropharyngeal LN
1171 (4 MPI)	3.04 × 10^3^	6.49 × 10^2^
1201 (4 MPI)	1.66 × 10^3^	1.09 × 10^3^
1215 (4 MPI)	9.60 × 10^3^	1.57 × 10^3^
1148 (2 MPI)	2.32 × 10^2^	3.95 × 10^1^
1157 (2 MPI)	4.00 × 10^2^	5.07 × 10^1^
